# Genome sequence of *Shimia* str. SK013, a representative of the *Roseobacter* group isolated from marine sediment

**DOI:** 10.1186/s40793-016-0143-0

**Published:** 2016-03-12

**Authors:** Saranya Kanukollu, Sonja Voget, Marion Pohlner, Verona Vandieken, Jörn Petersen, Nikos C. Kyrpides, Tanja Woyke, Nicole Shapiro, Markus Göker, Hans-Peter Klenk, Heribert Cypionka, Bert Engelen

**Affiliations:** Institute for Chemistry and Biology of the Marine Environment (ICBM), Oldenburg, Germany; Department of Genomic and Applied Microbiology and Göttingen Genomics Laboratory, Institute of Microbiology and Genetics, University of Göttingen, Göttingen, Germany; Leibniz Institute DSMZ – German Collection of Microorganisms and Cell Cultures, Braunschweig, Germany; Department of Energy Joint Genome Institute, Genome Biology Program, Walnut Creek, California USA; Department of Biological Sciences, Faculty of Science, King Abdulaziz University, Jeddah, Saudi Arabia; School of Biology, Newcastle University, Newcastle upon Tyne, UK

**Keywords:** Anaerobic metabolism, Cell-connecting filaments, Quorum quenching, Flagella gene cluster, DMSP, DMSO reductase, Denitrification

## Abstract

*Shimia* strain SK013 is an aerobic, Gram-negative, rod shaped alphaproteobacterium affiliated with the *Roseobacter* group within the family *Rhodobacteraceae*. The strain was isolated from surface sediment (0–1 cm) of the Skagerrak at 114 m below sea level. The 4,049,808 bp genome of *Shimia* str. SK013 comprises 3,981 protein-coding genes and 47 RNA genes. It contains one chromosome and no extrachromosomal elements. The genome analysis revealed the presence of genes for a dimethylsulfoniopropionate lyase, demethylase and the trimethylamine methyltransferase (*mttB*) as well as genes for nitrate, nitrite and dimethyl sulfoxide reduction. This indicates that *Shimia* str. SK013 is able to switch from aerobic to anaerobic metabolism and thus is capable of aerobic and anaerobic sulfur cycling at the seafloor. Among the ability to convert other sulfur compounds it has the genetic capacity to produce climatically active dimethyl sulfide. Growth on glutamate as a sole carbon source results in formation of cell-connecting filaments, a putative phenotypic adaptation of the surface-associated strain to the environmental conditions at the seafloor. Genome analysis revealed the presence of a flagellum (*fla*1) and a type IV pilus biogenesis, which is speculated to be a prerequisite for biofilm formation. This is also related to genes responsible for signalling such as N-acyl homoserine lactones, as well as quip-genes responsible for quorum quenching and antibiotic biosynthesis. Pairwise similarities of 16S rRNA genes (98.56 % sequence similarity to the next relative *S. haliotis*) and the *in silico* DNA-DNA hybridization (21.20 % sequence similarity to *S. haliotis*) indicated *Shimia* str. SK013 to be considered as a new species. The genome analysis of *Shimia* str. SK013 offered first insights into specific physiological and phenotypic adaptation mechanisms of *Roseobacter-*affiliated bacteria to the benthic environment.

## Introduction

The *Roseobacter* group is known for its worldwide distribution and its broad metabolic versatility in a great variety of marine habitats [1–3]. About 25 % of all *Roseobacter* species with validly published names (42 out of 168) have a benthic origin [4]. In marine sediments, they can contribute up to 11 of all 16S rRNA genes and up to 10 % of total cell counts [5, 6], but still little is known about the specific distribution and physiology of roseobacters in this habitat.

*Shimia* str. SK013, analysed in the present study, was isolated from the top centimeter of Skagerrak sediments at a water depth of 114 m below sea level (mbsl) [7]. The strain is affiliated with the genus *Shimia* which was first proposed by Choi and Cho in 2006 [8] in honor of Dr. Jae H. Shim, for his contributions to marine plankton ecology in Korea. According to Pujalte et al. [4], the genus *Shimia* consists of four species, with a fifth species *Shimia sagamensis* recently included. Members of the genus *Shimia* were isolated from different marine habitats: e.g. *S. haliotis* was isolated from the intestinal tract of the abalone *Haliotis discus hannai* [9], *S. biformata* from surface sea water [10], *S. isoporae* from reef building corals [11] and *S. marina* from a fish farm biofilm [8]. The new species affiliated to the genus *Shimia* (*Shimia sagamensis*) was isolated from cold seep sediment [12]. The sequenced genome of *Shimia* str. SK013 will allow for genetic comparison between the strain and other organisms of benthic origin, additional sediment-derived roseobacters and close relatives isolated from different habitats.

Here, we present the genome of *Shimia* str. SK013 with special emphasis on the genes involved in sulfur cycling such as dimethylsulfoniopropionate (DMSP) degradation and dimethyl sulfoxide reduction, as well as other anaerobic pathways such as nitrate reduction. The second focus is on genes which may be indicative for biofilm formation (pili, flagella and quorum sensing) as an adaptation to their surface-associated lifestyle.

## Organism information

### Classification and features

**Fig. 1:**
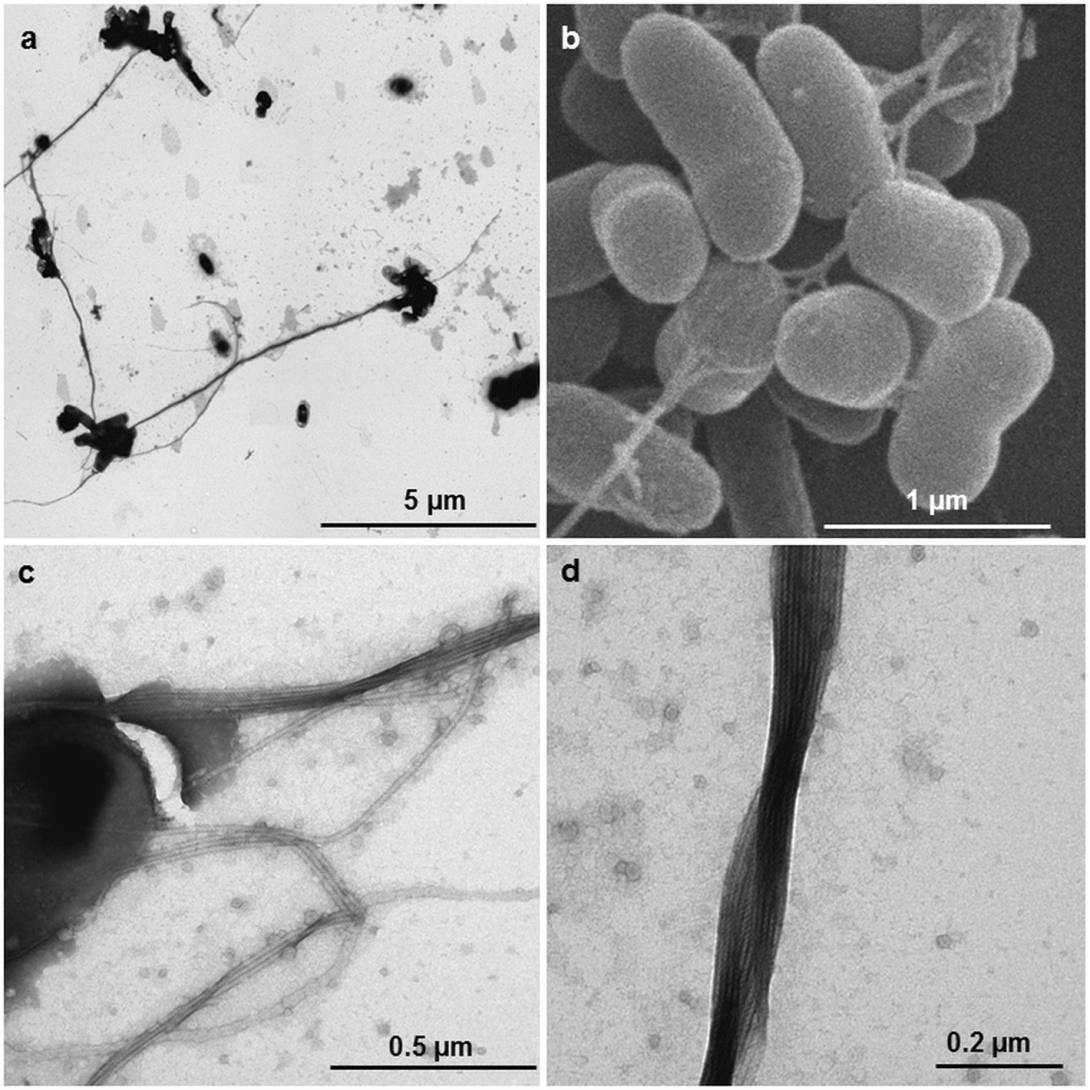
Micrographs of *Shimia* str. SK013. **a** Transmission electron micrograph (TEM) showing aggregation of cells and long fibers (**b**) Scanning electron micrograph (SEM) of cells grown on glutamate with formation of cell-connecting fibers (**c**) TEM of a single cell with cell-connecting fibers (**d**) Closer view (TEM) on a bundle of fibers

**Fig. 2:**
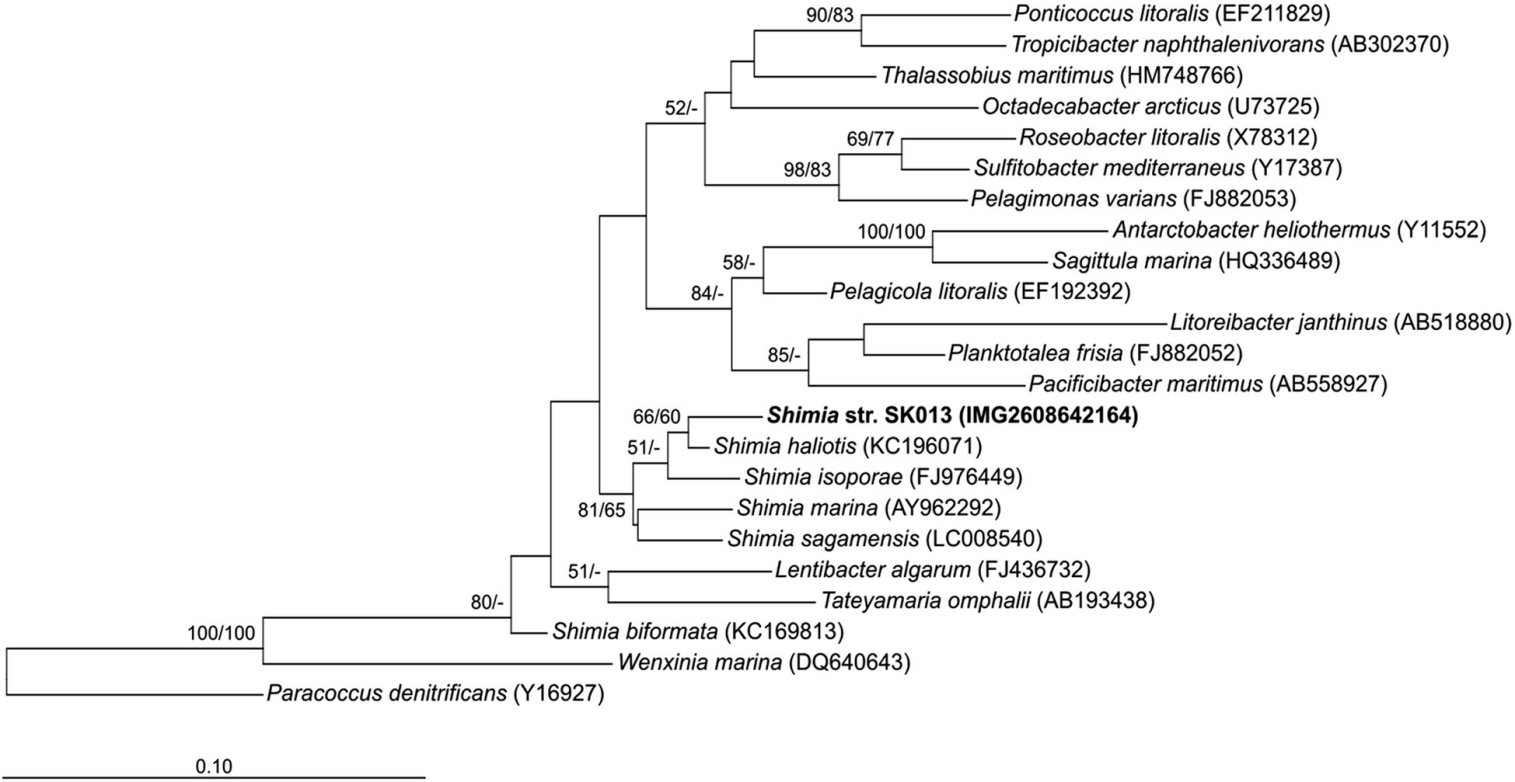
The 16S rRNA tree highlighting the position of *Shimia* str. SK013 relative to the other species within the genus *Shimia* and other type strains within the *Roseobacter* group. Maximum likelihood (ML; substitution model = GTR) tree, using 1453 aligned characters, was rooted by *Paracoccus denitrificans* another member of the *Rhodobacteraceae* family with ARB [12]. Branches were scaled in terms of the expected number of substitutions per site. Numbers adjacent to branches are support values from 1000 ML bootstrap replicates (*left*) and from 1000 maximum-parsimony bootstrap replicates (*right*); values below 50 % were neglected

Sediment samples were collected in July 2011 during a cruise with the RV ‘Heincke’ (expedition HE361) to the eastern North Sea. The strain was isolated from surface sediment (0–1 cm) of the Skagerrak (Site 27, 57°61.28′N, 8°58.18′E) at 114 mbsl from an aerobic enrichment culture. *Shimia* str. SK013 is a Gram-negative, motile, rod shaped bacterium with a length of 1.8 to 2.0 μm and a width of approximately 0.5 μm (Table 1; Fig. 1). Colonies are small, slightly domed and white to transparent on artificial sea water medium agar plates, but cream-coloured or beige in marine broth medium agar plates. The strain is mesophilic (range: 10–35 °C, T_opt_ = 30 °C), neutrophilic (optimum pH: 6–7) and halophilic (optimum: 2–3 % w/v). *Shimia* str. SK013 grows well in liquid medium but relatively slowly on agar-solidified marine broth and artificial sea water medium. The strain is able to utilize various substrates such as glucose, lactose, glutamate, mannose, xylose, acetate and citrate. When *Shimia* str. SK013 grows in ASW medium with glutamate as sole carbon source, cell-connecting filaments that might represent bundle-forming pili or specialized flagella are induced (Fig. 1). However, these structures were not observed in cultures amended with any other tested substrate (see above). The 16S rRNA gene sequence of *Shimia* str. SK013 (1453 bp) was analysed using ARB [13] and revealed 98.56 % sequence similarity to the next relative, *Shimia haliotis*. Furthermore, in the phylogenetic tree, *Shimia* str. SK013 is branching together with the other *Shimia* species except *Shimia biformata* (Fig. 2).

**Table 1:** Classification and general features of *Shimia* str. SK013 in accordance with the MIGS recommendations published by the Genome Standards Consortium [46]

MIGS ID	Property	Term	Evidence code^a^
	Classification	Domain *Bacteria*	TAS [47]
		Phylum *Proteobacteria*	TAS [48]
		Class *Alphaproteobacteria*	TAS [49, 50]
		Order *Rhodobacterales*	TAS [50, 51]
		Family *Rhodobacteraceae*	TAS [50, 51]
		Genus *Shimia*	TAS [8]
		Species *Shimia*	TAS [8]
		Strain SK013 (IMG2608642164)	TAS [7]
	Gram stain	negative	IDA
	Cell shape	Rod shaped	IDA
	Motility	Motile	IDA
	Sporulation	none	NAS
	Temperature range	Mesophile; 10–35 °C	IDA
	Optimum temperature	25–30 °C	IDA
	pH range; Optimum	5–9; 7	IDA
	Carbon source	Sugars, amino acids	IDA
MIGS-6	Habitat	Marine	IDA
MIGS-6.3	Salinity	0–5 % NaCl (w/v)	IDA
MIGS-22	Oxygen requirement	Aerobic	IDA
MIGS-15	Biotic relationship	Unknown	NAS
MIGS-14	Pathogenicity	non-pathogen	NAS
MIGS-4	Geographic location	North Sea/Skagerrak area	IDA
MIGS-5	Sample collection	July 24, 2011	IDA
MIGS-4.1	Latitude	57°36.77‘N	IDA
MIGS-4.2	Longitude	08°35.41‘E	IDA
MIGS-4.3	Depth	114 m below sea level	IDA
MIGS-4.4	Altitude	Unknown	

^a^Evidence codes - *IDA* Inferred from Direct Assay, *TAS* Traceable Author Statement (i.e., a direct report exists in the literature), *NAS* Non-traceable Author Statement (i.e., not directly observed for the living, isolated sample, but based on a generally accepted property for the species, or anecdotal evidence). These evidence codes are from the Gene Ontology project [52]

## Genome sequencing information

### Genome project history

*Shimia* str. SK013 was selected for draft genome sequencing based on its physiological and phenotypical features and its benthic origin. The information related to this project is summarized in Table 2. The draft genome is deposited in the Genomes On Line Database [14] and in the Integrated Microbial Genome database [15]. The Whole Genome Shotgun project has been deposited at DDBJ/EMBL/GenBank under the accession number LAJH00000000.1.

**Table 2:** Genome sequencing project information

MIGS ID	Property	Term
MIGS 31	Finishing quality	Draft
MIGS-28	Libraries used	Nextera xt
MIGS 29	Sequencing platforms	Illumina GAii, PacBio
MIGS 31.2	Fold coverage	
MIGS 30	Assemblers	SPAdes v3.5
MIGS 32	Gene calling method	Prodigal v2.5
	Locus Tag	SHIM
	Genbank ID	LAJH00000000
	GenBank Date of Release	September 16, 2015
	GOLD ID	Gp0103193
	BIOPROJECT	PRJNA277163
MIGS 13	Source Material Identifier	SAMN03387008
	Project relevance	Environmental

### Growth conditions and genomic DNA preparation

*Shimia* str. SK013 was enriched and isolated from agar plates containing artificial sea water medium [16] with DMS (100 μM) and lactate (5 mM) as substrates, incubated at 15 °C. The genomic DNA extraction was performed using a DNA isolation kit (MO BIO, Carlsbad, CA, USA), following the manufactures instructions.

### Genome sequencing and assembly

Whole-genome sequencing was performed using the Illumina technology. Preparation of paired-end sequencing library with the Illumina Nextera XT library preparation kit and sequencing of the library using the Genome Analyzer IIx were performed as described by the manufacturer (Illumina, San Diego, CA, USA). A total of 11,098,582 paired-end reads were derived from sequencing and trimmed using Trimmomatic version 0.32 [17]. *De novo* assembly of all trimmed reads with SPAdes version 3.5.0 [18] resulted in 28 contigs and 137.9-fold coverage. A summary of project information is shown in Table 2.

### Genome annotation

Protein-coding genes were identified as part of the genome annotation pipeline the Integrated Microbial Genomes Expert Review platform using Prodigal v2.50. The predicted CDS were translated and used to search the CDD, KEGG, UniProt, TIGRFam, Pfam and InterPro databases. These data sources were combined to assert a product description for each predicted protein. Non-coding genes and miscellaneous features were predicted using tRNAscan-SE [19], RNAmmer [20], Rfam [21], TMHMM [22] and SignalP [23]. Additional gene prediction analyses and functional annotation were performed within the IMG-Expert Review platform [24].

## Genome properties

**Fig. 3:**
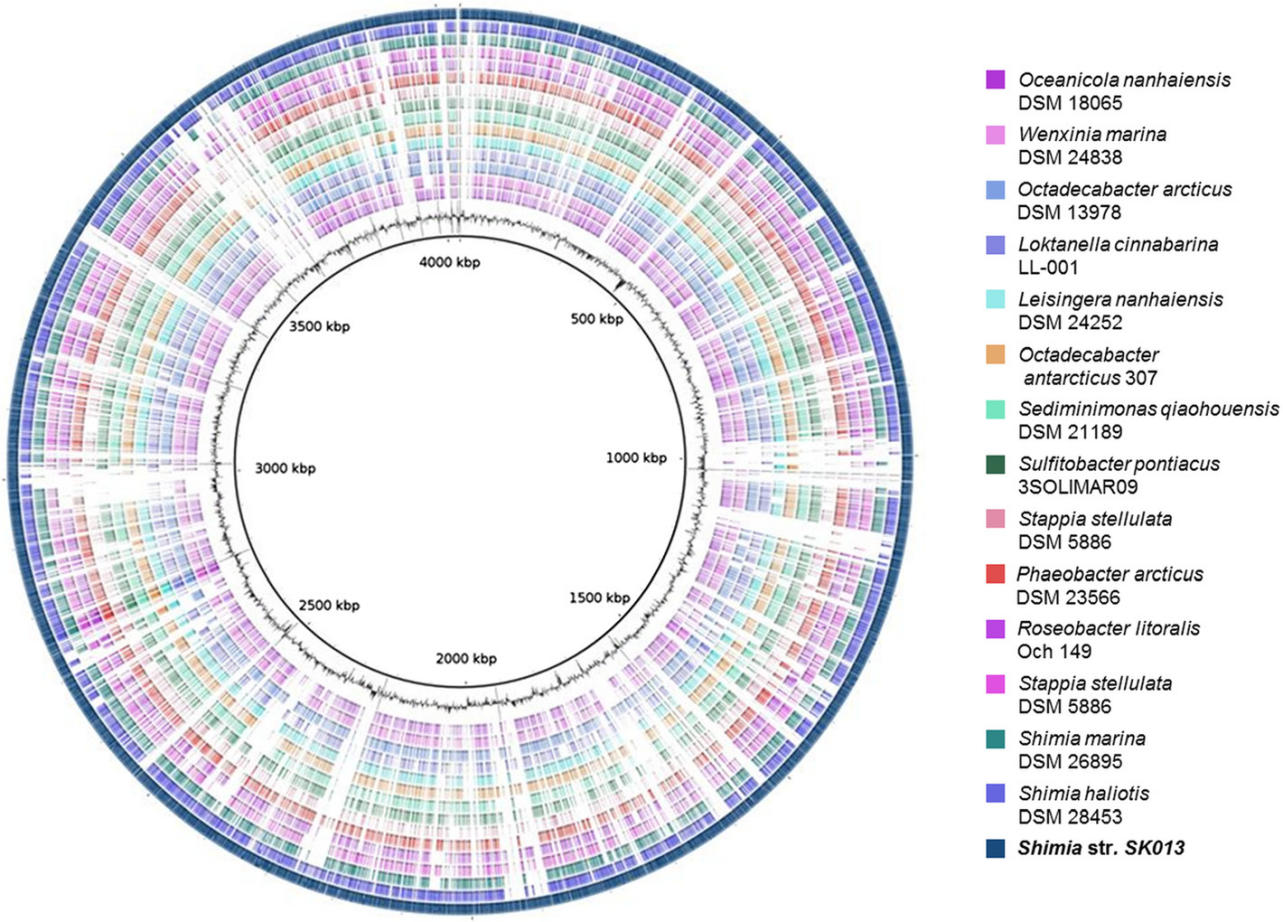
Graphical representation of the genome of *Shimia* str. SK013. From outside to inside (1–15 color circles): sequence of *Shimia* str. SK013 (1^st^ circle) is compared to the other species within the genus *Shimia* and other type strains within the *Roseobacter* group, (16^th^ circle): G + C content of *Shimia* str. SK013. Comparisons and visualizations are performed with BRIG [53]

The genome analysis showed the presence of 28 scaffolds corresponding to one large chromosome with a total length of 4,049,808 bp and a G + C content of 57.22 % (Table 3). The absence of additional extrachromosomal elements was inferred based on the absence of RepABC, RepA, RepB and DnaA-like modules for plasmid replication and maintenance that are characteristic for *Rhodobacteraceae* [25]. In total, 4,028 genes were predicted, in which 3,981 were protein-coding genes and 47 RNA genes. About 82.35 % were protein-coding genes with a putative function while those remaining were annotated as hypothetical proteins. The genome statistics are further provided in Table 3 and in Fig. 3. The distribution of genes into functional categories (clusters of orthologous groups) is shown in Table 4.

**Table 3:** Genome statistics of *Shimia* str. SK013

Attribute	Value	% of total
Genome size (bp)	4,049,808	100.00
DNA coding (bp)	3,677,855	90.82
DNA G + C (bp)	2,317,341	57.22
DNA scaffolds	28	
Total genes	4028	100.00
Protein-coding genes	3981	98.83
RNA genes	47	1.17
Pseudo genes	0	
Genes in paralog clusters	3069	76.19
Genes with function prediction	3317	82.35
Genes assigned to COGs	2860	71.00
Genes with Pfam domains	3365	83.54
Genes with signal peptides	370	9.19
Genes with transmembrane helices	911	22.62
CRISPR repeats	0

**Table 4:** Number of genes associated with general COG functional categories

Code	Value	%age	Description
J	194	6.02	Translation, ribosomal structure and biogenesis
A	n.a.	n.a.	RNA processing and modification
K	221	6.86	Transcription
L	90	2.80	Replication, recombination and repair
B	2	0.06	Chromatin structure and dynamics
D	25	0.78	Cell cycle control, Cell division, chromosome partitioning
V	61	1.89	Defense mechanisms
T	126	3.91	Signal transduction mechanisms
M	178	5.53	Cell wall/membrane biogenesis
N	50	1.55	Cell motility
U	41	1.27	Intracellular trafficking and secretion
O	156	4.84	Posttranslational modification, protein turnover, chaperones
C	238	7.39	Energy production and conversion
G	203	6.30	Carbohydrate transport and metabolism
E	388	12.05	Amino acid transport and metabolism
F	87	2.70	Nucleotide transport and metabolism
H	174	5.40	Coenzyme transport and metabolism
I	186	5.78	Lipid transport and metabolism
P	143	4.44	Inorganic ion transport and metabolism
Q	130	4.04	Secondary metabolites biosynthesis, transport and catabolism
R	313	9.72	General function prediction only
S	192	5.96	Function unknown
-	1168	29.00	Not in COGs

The total is based on the total number of protein-coding genes in the genome

## Insights from the genome sequence

The genome of *Shimia* str. SK013 contains genes for sulfur cycling that might enable anaerobic growth. Genes for quorum sensing and quorum quenching might support roseobacters to thrive in complex microbial communities found in sediments. Genome comparison (Table 5; Fig. 3) revealed that *Shimia* str. SK013 shares the respective genes with a selection of surface-associated roseobacters and the other two *Shimia* species whose genomes are available. It is well documented that roseobacters are involved in the transformation of DMSP by demethylation or by using the cleavage pathway [6, 26, 27]. Anaerobically, some roseobacters are capable of DMSO reduction resulting in the release of the climatically active DMS [5, 27]. Genes for the DMSP lyase (Shim_05930) and demethylase (Shim_7490) as well as for the DMSO reductase (Shim_34610) found in *Shimia* str. SK013 indicate their functional role in DMSP degradation and DMSO conversion. All three genes are also present within the genome of *S. haliotis* and in those of a selection of surface-associated roseobacters (Table 6): *S. haliotis* (Ga0070219_1011011, 101620, 103192), *Octadecabacter arcticus* (OA238_c10540, c20430, c35930), *Roseobacter litoralis* (RLO149_c019880, c022350, c001820) and *Leisingera nanhaiensis* (Leina_00726, 01164, 02539). *Shimia marina* is missing the genes for DMSP lyase, but also contains genes for DMSP demethylase (Ga0069993_10296, 102173) and DMSO reductase (Ga0069993_106210). Interestingly, the genome of *Shimia* str. SK013 simultaneously contained genes for a sulfite reductase (Shim_12650), sulfur dehydgrogenase (*SoxC*; Shim_11330), sulfur oxidizing proteins (*SoxXYZ*; Shim_11380, 11370, 11360) and sulfur oxidation (*SoxA*; Shim_11350). Other than in the *Roseobacter* group-affiliated fosmid found in German tidal-flat sediments [6], the *soxD* gene and the rDSR gene are not present.

**Table 5:** Genome statistics comparison with available genomes of *Shimia* species

Genome name	*Shimia str.* SK013	*S. haliotis*	*S. marina*
		DSM 28453	DSM 26895
Genome Size	4,049,808	3,995,969	4,061,252
Gene Count	4,028	3,953	3,992
Scaffold Count	28	22	32
G + C content (%)	57.22	58.04	57.34
RNA Count	47	58	61
rRNA Count	3	5	5
COG Count	2,860	2,751	2,776
COG (%)	71.00	69.59	69.54
Pfam Count	3,350	3,300	3,365
Pfam (%)	83.17	83.48	84.29
TIGRfam Count	1,148	1,155	1,172
TIGRfam (%)	28.50	29.22	29.36
IMG Pathway Count	223	213	207
IMG Pathway (%)	5.54	5.39	5.19
Horizontally Transferred Count	223	158	135
Horizontally Transferred (%)	5.54	4.00	3.38

**Table 6:** Highlighted genes of *Shimia* str. SK013 present in other roseobacters

Highlighted gene products and locus tags	DMSP lyase DddP Shim_05930	DMSP Demethylase Shim_07490	DMSO reductase Shim_34610	Trimethylamine methyltransferase Shim_09600, 31260	Type IV pilus biogenesis Shim_13020	AHL acylase QuiP precursor Shim_09300	Homoserine/homoserine lactone efflux protein Shim_16180	N-AHLs Shim_31370
*Shimia haliotis*	+	+	+	+	+	+	+	+
*Shimia marina*		+	+	+	+	+	+	
*Oceanicola nanhaiensis*	+			+			+	+
*Octadecabacter antarcticus*		+	+	+		+		
*Octadecabacter arcticus*	+	+	+	+		+		
*Roseobacter litoralis*	+	+	+	+		+	+	+
*Phaeobacter arcticus*		+	+	+	+	+	+	
*Stappia stellulata*				+			+	
*Leisingera nanhaiensis*	+	+	+		+	+		
*Labrenzia aggregata*								
*Loktanella cinnabarina*				+		+		+
*Sulfitobacter pontiacus*				+	+	+		
*Sediminimonas qiaohouensis*		+		+		+	+	+
*Wenxinia marina*				+		+	+	

We observed all genes necessary for the denitrification pathway such as nitrate reductase (Shim_01900), nitrite reductase (Shim_01920), nitric oxide reductase (Shim_02650) and nitrous oxide reductase (Shim_02640). *Shimia* str. SK013 contains a periplasmic nitrate reductase composed of five subunits [28] such as NapA (Shim_18270), NapB (Shim_18300), NapD (Shim_18260), NapE (Shim_04260) and NapG (Shim_18280). The presence of periplasmic nitrate reductase genes suggest the potential for anaerobic respiration [29] in *Shimia* str. SK013, whereas the genus *Shimia* has been described as strictly aerobic until now [8]. Interestingly, anaerobic respiration was also observed in *Leisingera nanhaiensis* [30] and *Phaeobacter inhibens* T5^T^ [31], which were originally described as strictly aerobic. The genes involved in nitrogen regulation (Shim_09380) and nitrogen fixation regulation (Shim_29520) were also found in the genome of *Shimia* str. SK013. Denitrification genes in *Shimia* str. SK013 showed a strong resemblance to those present in *S. haliotis*, with the exception the genes coding for nitrite reductase and nitrogen fixation regulation (nitrate reductase and subunits; Ga0070219_10142 to 10145, nitric oxide reductase; Ga0070219_106169; nitrous oxide reductase; Ga0070219_106170, nitrogen regulation; Ga0070219_101812). *S. marina* showed only the presence of genes for nitrate reduction (Ga0069993_10650), nitrite reduction (Ga0069993_10648), nitrogen regulation (Ga0069993_102260) and nitrogen fixation regulation (Ga0069993_105163). A comparative search revealed the presence of all the genes involved in the nitrogen cycle that were mentioned above for *Oceanicola nanhaiensis* (SIAM614_16412, 31426, 14520, 22007), *Roseobacter litoralis* (RLO149_c039850, c031550, c017950, c035140), *Phaeobacter arcticus* (Phaar_03838, 02837, 01419, 03079, 04163) and *Sulfitobacter pontiacus* (PM01_06655, 15855, 12625, 02530). Furthermore, the genome of *Shimia* str. SK013 revealed genes for the utilization of methylated amines, such as a trimethylamine methyltransferase (*mttB*) (Shim_09600, 31260).

The conspicuous morphological trait of cell-connecting filaments in *Shimia* str. SK013 (Fig. 1) led to the search for the presence of genes involved in the formation of pili and flagella. The bacterial flagellum is one of the signal transduction systems with complex proteins which enables the bacterial reorientation and motility [32]. So far three different types of flagella gene clusters (FGCs) were described, designated *fla1*, *fla2* and *fla*3 in *Rhodobacteraceae* that originated from FGC duplications [33]. Genome analysis revealed the presence of a single compact flagella gene cluster of the *fla1*-type on the chromosome (contig_000021; Shim_33080 to Shim_33420) that contains all genes necessary for the assembly of a functional flagellum. Recently, Frank et al. [33] showed for the plasmid curing mutant of *Marinovum algicola* DG898, which is lacking the 143-kb plasmid pMaD5 with a *fla2*-type FGC, a conspicuous morphological similarity with the filamentous structures observed in the current study for *Shimia* str. SK013 (Fig. 1). The bundles of filaments were explained by the presence of an additional chromosome-encoded *fla1*-type flagellum in *Marinovum*. However, genes for type IV pilus biogenesis, which were found in *Shimia* str. SK013 (Shim_13020, Shim_37620) are also present in the genome of *M. algicola* DG898 (MALG_02262) and thus, it is remains unclear if the conspicuous bundles at the cell pole are caused by pilus and/or flagellum formation.

As the described morphological traits are often related to a surface-associated lifestyle, we also searched the genome of *Shimia* str. SK013 for genes involved in the production of signalling molecules and quorum sensing as indicators for the communication within biofilms. Earlier studies showed that quorum sensing signals are mainly associated with virulence [34, 35], but recent investigations revealed that these signalling molecules play a significant role in basic metabolic processes [36, 37]. The presence of genes for the production of *N*-acylhomoserine lactones (AHLs) (Shim_31370) and homoserine lactones (Shim_16180) that are part of the quorum sensing system indicate that *Shimia* str. SK013 uses this form of bacterial communication. In contrast, the newly established genome only contains a few additional genes which interfere with quorum sensing such as quorum quenching or antibiotic biosynthesis related genes (AHL acylase QuiP precursor; Shim_09300) [38–40]. When compared to other selected roseobacters, these three signal molecule genes were also found in *Roseobacter litoralis* (RLO149_c018030*,* c029420, c006500) and *Sediminimonas**qiahouensis* (G568DRAFT_00799, 01106, 03483). This finding was proven by an antiSMASH analysis [41] of the *Shimia* str. SK013 genome, indicating the presence of the type I polyketide synthase (PKS), the homoserine lactone cluster and the bacteriocin gene cluster.

Pairwise similarities of 16S rRNA genes of *Shimia* str. SK013 and the next relative, *Shimia haliotis* were 98.56 %. A genome comparison of *Shimia* str. SK013 with the available draft genomes from the KMG-2 project, Genomic encyclopedia of Bacteria and Archaea (GEBA) [42, 43] of *Shimia haliotis*DSM 28453 (IMG ID: 2619619046) and *Shimia marina*DSM 26895 (IMG ID: 2619618961) was conducted using the online analysis tool “Genome-Genome-Distance Calculator” 2.0 (GGDC). The results of the *in silico* calculated DNA-DNA hybridization (DDH) of *Shimia* str. SK013 suggests that the given genome might belong to a new species based on the low percentages obtained (Table 7). According to the GGDC tool, formula 2 was recommended for the comparison between the draft genomes as it provides higher DDH correlations than Average Nucleotide Identity (ANI) implementations [44, 45]. The analysis showed that *Shimia* str. SK013 only shared a genome sequence similarity of 21 % with *Shimia haliotis*DSM 28453 and 20 % with *Shimia marina*DSM 26895 and thus represents neither a new isolate of the species *S. haliotis* nor of *S. marina*. A direct comparison with the available *Shimia* genomes revealed further differences such as the IMG pathway counts (representing the number of metabolites and macromolecular complexes) and horizontally transferred gene counts (Table 5). Until now, genome sequences of *S. bioformata*, *S. isoporae* and *Shimia sagamensis* are not available for additional *in silico* calculated DNA-DNA hybridization or direct genome comparisons. However, as *S. haliotis* was identified as the closest relative by 16S rRNA gene analysis with a 66/60 % bootstrap support, the DDH data provide strong evidence that *Shimia* str. SK013 represents a new species within the genus *Shimia*.

**Table 7:** Digital DDH similarities between *Shimia* str. SK013 and other *Shimia* species, calculated *in silico* with the GGDC server version 2.0 [45]^a^

Reference species	Formula 1	Formula 2	Formula 3
*Shimia haliotis* DSM 28453	37.20 % +/− 3.44	21.20 % +/− 2.34	31.60 % +/− 3.02
*Shimia marina* DSM 26895	16.70 % +/− 3.25	19.70 % +/− 2.30	16.60 % +/− 2.75

^a^The standard deviations indicate the inherent uncertainty in estimating DDH values from intergenomic distances based on models derived from empirical test data sets (which are always limited in size); see [45] for details. The distance formulas are explained in [44]. Formula 2 is recommended, particularly for draft genome (like species above)

## Conclusions

The genome analysis of *Shimia* str. SK013 revealed distinctive genes responsible for DMSP utilization, DMSO, nitrate and nitrite reduction which indicate that this strain is a facultative anaerobic bacterium. The presence of genes responsible for signalling can serve as a guide for identification of quorum sensing compounds, as well as antibiotics potentially responsible for quorum quenching. Based on genome comparison and DNA-DNA hybridization with the next relatives, *Shimia* str. SK013 might represent a new species and should be considered for species description.

## Abbreviations

AHLs: acyl homoserine lactones
ASW: artificial sea water
BFP: bundle-forming pili
DMS: dimethyl sulfide
DMSO: dimethyl sulfoxide
DMSP: dimethylsulfoniopropionate
FGC: flagella gene clusters
GGDC: Genome-Genome-Distance Calculator
KMG: 1000 microbial genomes
mbsl: meters below sea level
PKS: polyketide synthase
SignalP: signal peptides
TMHMM: transmembrane helices hidden markov models
